# Genotype-phenotype correlation of renal lesions in the tuberous sclerosis complex

**DOI:** 10.1038/s41439-022-00181-1

**Published:** 2022-02-10

**Authors:** Yoshinari Muto, Hitomi Sasaki, Makoto Sumitomo, Hidehito Inagaki, Maki Kato, Takema Kato, Shunsuke Miyai, Hiroki Kurahashi, Ryoichi Shiroki

**Affiliations:** 1 Department of Urology, School of Medicine, Fujita Health University, Toyoake, Japan; 2 Division of Molecular Genetics, Institute for Comprehensive Medical Science, Fujita Health University, Toyoake, Aichi 470-1192 Japan

**Keywords:** DNA sequencing, Urogenital diseases, Genetics research

## Abstract

Tuberous sclerosis complex (TSC) is an autosomal dominant disease caused by loss-of-function mutations in either of two tumor suppressor genes, *TSC1* and *TSC2*. These mutations lead to the growth of benign tumors and hamartomas in many organs, including those of the central nervous system, the skin, and the kidneys. To investigate the genotype-phenotype correlation, we performed sequence analysis of the *TSC1*/*2* genes using next-generation sequencing. We classified 30 patients with TSC whose pathogenic variants were identified into two groups: those with mutations producing premature termination codons (PTCs) and those with missense mutations. Then, we compared the phenotypes between the two groups. Patients with a PTC were significantly more likely to manifest the major symptoms of the diagnostic criteria than those without a PTC (*P* = 0.035). The frequencies of subependymal nodules (*P* = 0.026), cortical tubers (*P* = 0.026), and renal cysts (*P* = 0.026) were significantly higher in PTC-containing variants than in cases without a PTC. When the analyses were limited to renal angiomyolipoma (AML) cases with *TSC2* mutations, there was no difference in tumor size between cases with and without a PTC. However, the cases with a PTC showed a trend toward disease onset at a younger age and multiple tumors, and bilateral disease was observed in their AML lesions. TSC patients with PTC-producing mutations might potentially manifest more severe TSC phenotypes than those with missense mutations. A larger-scale study with appropriate samples deserves further investigation.

## Tuberous sclerosis: type of mutation affects disease presentation

The ways in which a rare genetic disorder called tuberous sclerosis complex presents depends on the type of genetic mutation involved. Tuberous sclerosis is characterized by non-cancerous growths that form in many parts of the body. Yoshinari Muto and colleagues from Fujita Health University in Toyoake, Japan, sequenced the two genes linked to most cases of the disease, using blood samples from 30 patients. People with mutations that created a premature stop signal during protein synthesis were more likely to develop growths in the brain, skin, and kidney compared to people with mutations involving just one amino acid in a protein sequence. Patients with early stop–type genetic defects tended to exhibit symptoms at a younger age and have more kidney growths. The findings suggest that genetic diagnoses could help predict disease severity and guide clinical decision-making.

## Introduction

Tuberous sclerosis complex (TSC) is an autosomal dominant genetic disease affecting one in every 6,000–10,000 live births^1^. It is mainly characterized by the growth of benign tumors and other hamartoma lesions in various organs throughout the body, including the kidney, brain, lung, skin, and heart^2^. Renal angiomyolipoma (AML) is a pathologically benign mesenchymal kidney tumor characterized by vascular smooth muscle and adipocyte elements^3^. AMLs are rare in the general population (autopsy prevalence of ~1 in 1,000). However, they affect >70% of adults with TSC, where they are usually multifocal and bilateral^2^. In fact, for those with TSC, acute bleeding of renal AML caused by spontaneous or traumatic rupture is the main cause of death^4^. Moreover, pathogenic mutations in *TSC1* and *TSC2* are found in 75–90% of cases that meet the standard clinical criteria for TSC^5,6^. The *TSC1* gene is located at 9q34 and contains 23 exons. It is 50 kb in length and codes for the hamartin protein (molecular weight, 130 kDa, 1164 amino acids). The *TSC2* gene is located at 16p13.3 and contains 42 exons. It is 45 kb in length and codes for the tuberin protein (molecular weight, 190 kDa, 1807 amino acids)^7^. Approximately 60–70% of TSC cases are sporadic, which reflects a high *de novo* mutation rate in these genes^8^.

Previous genetic studies have shown that renal AML occurring in TSC cases was caused by the inactivation of both pairs of alleles of either *TSC1* or *TSC2*, which is consistent with Knudson’s 2-hit tumor suppressor gene model^2,5,9–12^. TSC1 (hamartin) and TSC2 (tuberin) complexes act as inhibitors of the mTOR pathway^10,11,13,14^. Biallelic mutations of *TSC1* or *TSC2* activate the mTOR pathway, leading to an advantage for the growth of affected cells. mTOR inhibitors have been demonstrated to be effective in suppressing the growth of TSC tumors^14^.

Pathogenic mutations in the *TSC1* or *TSC2* genes are often nonsense or frameshift mutations that lead to premature termination codons (PTCs)^10^. When mRNA contains a PTC, cells utilize an mRNA decay pathway, called nonsense-mediated mRNA decay (NMD), to eliminate nonfunctional transcripts^15^. Alleles with nonsense or frameshift mutations do not produce any protein. However, alleles with missense mutations produce proteins with partially altered amino acid sequences that can affect protein stability, hydrogen bonding, dynamics, and activity, leading to alterations in protein function^16^. Thus, it is possible that the type of mutations in the *TSC1* or *TSC2* gene could affect the clinical phenotypes of the patient. The purpose of this study was to clarify the significance of germline mutations in the characteristics of renal AML in TSC cases.

## Materials and methods

### Patients

This study was approved by the local Ethical Review Committee of Fujita Health University (HG-20-054). Written informed consent was obtained from all 30 patients with TSC included in this study. These patients were located at Fujita Health University and were clinically diagnosed with TSC mainly by clinical features and imaging evaluation according to the latest diagnostic criteria^17^. The median age of the patients was 24 years (range, 14–28 years). There were 12 male (40%) and 18 female (60%) patients (Table 1). The age at onset of AML was the age of the patient when they were first diagnosed with renal AML by computed tomography (CT), and the maximum diameter of the AML was measured on CT.

**Table 1:** Phenotypic characteristics of patients with tuberous sclerosis complex in this study.

Characteristic		All (*N* = 30)	*TSC1* (*N* = 4)	*TSC2* (*N* = 23)	NMI (*N* = 3)
Median age, years (range)		24 (14–28)	21.5 (16.3–28)	22 (11.3–27.3)	34 (30.5–39.5)
Gender	Male (%)	12 (40.0)	1 (25.0)	11 (47.8)	0 (0)
	Female (%)	18 (60.0)	3 (75.0)	12 (52.1)	3 (100)
Hypomelanotic macules (%)		9 (30.0)	2 (50.0)	6 (26.1)	1 (33.3)
Angiofibromas (%)		20 (66.7)	1 (25.0)	17 (73.9)	2 (66.7)
Ungual fibromas (%)		9 (30.0)	1 (25.0)	7 (30.4)	1 (33.3)
Shagreen patch (%)		5 (17.2)	1 (33.3)	4 (17.4)	0 (0)
Multiple retinal hamartomas (%)		7 (23.3)	0 (0)	7 (30.4)	0 (0)
Cortical tuber (%)		26 (86.7)	4 (100)	21 (91.3)	1 (33.3)
SEN (%)		26 (86.7)	4 (100)	21 (91.3)	1 (33.3)
SEGA (%)		4 (13.3)	1 (25.0)	3 (13.0)	0 (0)
Cardiac rhabdomyoma (%)		6 (20.0)	0 (0)	6 (26.1)	0 (0)
LAM (%)		11 (36.7)	1 (25.0)	8 (34.8)	2 (66.7)
AML (%)		26 (86.7)	3 (75.0)	20 (87.0)	3 (100)
Renal cyst (%)		12 (40.0)	4 (100)	8 (34.8)	0 (0)
AML maximum diameter, cm, median (range)		3.6 (2.0–8.9)	1.7 (0.8–3.7)	3.4 (2.1–8.9)	9 (8.7–10.3)

*AML* angiomyolipoma, *LAM* Lymphangiomyomatosis, *NMI* no mutation identified, *SEGA* subependymal giant cell astrocytoma, *SEN* subependymal nodule.

In the clinical phenotypes of these patients, 26 patients (89.7%) had renal AML, 26 patients (89.7%) had subependymal nodules, 27 (91.3%) had cortical tubers, 4 (13.8%) had subependymal giant cell astrocytomas, and 16 (55.2%) experienced seizures. Additionally, 11 patients (37.9%) presented with LAM, and 22 patients (75.9%) exhibited skin lesions (Table 1).

### Mutation analysis

Peripheral blood samples were collected from each patient, and genomic DNA was isolated from peripheral leukocytes (Qiagen, Frankfurt, Germany). The *TSC1* and *TSC2* genomic regions were amplified using long-range polymerase chain reaction (PCR). The Nextera XT DNA Library Preparation Kit (Illumina, San Diego, CA, USA) was used to fragment the genomic DNA following the manufacturer’s instructions. Then, AMPure XP (Beckman Coulter, Pasadena, CA, USA) was used to purify the library DNA and to remove short library fragments. Amplification with DNA polymerase and sequencing of the DNA samples were performed using Illumina MiSeq. Sequence reads were then mapped to a human reference sequence (RefSeq: NM_003639.3), and the identified sequence variants were confirmed by Sanger sequencing. The pathogenicity of the variants was evaluated by standard criteria^18^.

### Phenotype-genotype correlation

To analyze the effect of genetic mutations on the phenotypic appearance in patients with TSC, splicing site mutations were excluded. The frequency of each phenotype was compared between the patients with mutations producing PTC (nonsense mutation or frameshift mutation) and those with missense mutations.

### Statistical analysis

Continuous variables between the two groups were compared by the Mann–Whitney *U*-test. Proportions between the two groups were compared by Fisher’s test, and the *P*-value was calculated. *P*-values < 0.05 were considered statistically significant. Statistical analysis was conducted using the EZR software package (R version 3.4.1, Saitama Medical Center/Jichi Medical University, Saitama, Japan)^19^, which is a graphical user interface for R version 3.4.1 (R Foundation for Statistical Computing, Vienna, Austria).

## Results

Of the 30 patients enrolled in this study, 23 had mutations in *TSC2*, four had mutations in *TSC1*, and no mutation was found in the remaining three patients (Table 1).

**Fig. 1:**
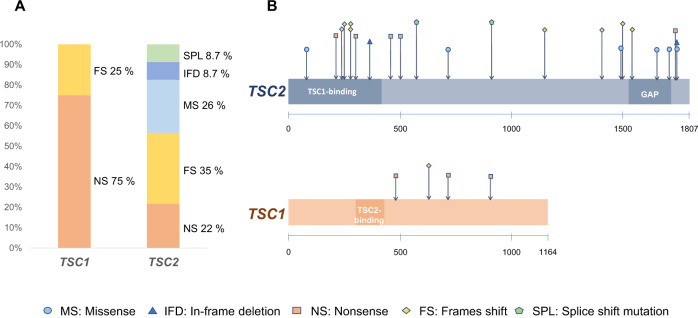
Type and distribution of germline mutations in *TSC1/TSC2*. **A** Percentage of germline mutation types in *TSC1/TSC2*. **B** Distribution of *TSC1*/*TSC2* mutations.

Of the four cases with *TSC1* mutations, three had nonsense mutations, and the other had a frameshift mutation. Among the 23 cases with *TSC2* mutations, five had nonsense mutations, eight had frameshift mutations, six had missense mutations, two had in-frame deletions, and two had splice site mutations. All of the mutations in *TSC1* and the 13 mutations in *TSC2* (56.5%) resulted in PTC (Fig. 1A). Moreover, no mutation hotspots were observed, and the mutations were located throughout the coding region of the genes (Fig. 1B).

To determine the effect of the type of mutations in *TSC1* or *TSC2* genes on the clinical course of the patient, we analyzed the correlation between the type of mutation, PTC or missense mutation, and the clinical features of the patients. Patients with a PTC were significantly more likely to manifest the major symptoms of the diagnostic criteria than those without a PTC (Table 2)^17^. Among the clinical features (Table 2), hypomelanotic macules, subependymal nodules (SENs), and cortical tubers were significantly more frequent in cases with a PTC (69.2% vs. 100%, *P* = 0.026), while all the other features did not show any significant differences between PTC and missense mutations. For the renal phenotype, only the frequency of renal cysts was found to be significantly more frequent in cases with PTC mutations (15.4% vs. 58.8, *P* = 0.026) (Table 3). Indeed, the maximum diameter of AML ranged from 2–8.9 cm (median 3.6 cm), and no difference was observed between the presence and absence of PTC mutations (Table 1 and Table 3). However, the median maximum diameter of *TSC1* AML was 1.7 cm (0.8–3.7 cm), while that of *TSC2* was 3.4 cm (2.1–8.9 cm). These results suggest that the differences in the causative genes might affect the clinical features, although the differences were not statistically significant (Table 1). Thus, we analyzed the phenotype-genotype correlation in kidney AML lesions, focusing only on patients with *TSC2* mutations.

**Table 2:** Observed frequencies of clinical features for all patients in this study.

	PTC (−) (*N* = 13)	PTC (+) (*N* = 17)	*P*-value
Hypomelanotic macules (%)	1 (7.7)	8 (47.1)	0.041
Angiofibromas (%)	8 (61.5)	12 (70.6)	0.705
Ungual fibromas (%)	3 (23.1)	6 (35.3)	0.691
Shagreen patch (%)	2 (15.4)	3 (18.8)	1
Multiple retinal hamartomas (%)	2 (15.4)	5 (29.4)	0.427
Cortical tuber (%)	9 (69.2)	17 (100)	0.026
SEN (%)	9 (69.2)	17 (100)	0.026
SEGA (%)	1 (7.7)	3 (17.6)	0.613
Cardiac rhabdomyoma (%)	3 (23.1)	3 (17.6)	1
LAM (%)	4 (30.8)	7 (41.2)	0.708
AML (%)	11 (84.6)	15 (88.2)	1

*AML* angiomyolipoma, *LAM* Lymphangiomyomatosis, *PTC* premature termination codon, *SEGA* subependymal giant cell astrocytoma, *SEN* subependymal nodule.

**Table 3:** Observed frequencies of renal clinical features for all patients in this study.

	PTC (−) (*N* = 13)	PTC (+) (*N* = 17)	*P*-value
Maximum diameter of AML, cm	5.5 (2.6–9.0)	3.4 (2.0–7.5)	0.722
Age at onset of AML, years	27 (16–30)	21 (14.8–28)	0.621
Bilateral AML (%)	7 (53.8)	11 (64.7)	0.711
Multiple AML (%)	9 (69.2)	11 (64.7)	1
Renal cyst (%)	2 (15.4)	10 (58.8)	0.026
Bilateral cyst (%)*	2 (100)	7 (70)	1
Multiple cyst (%)*	2 (100)	6 (60)	1

*Percent within the cases with renal cysts.

**Fig. 2:**
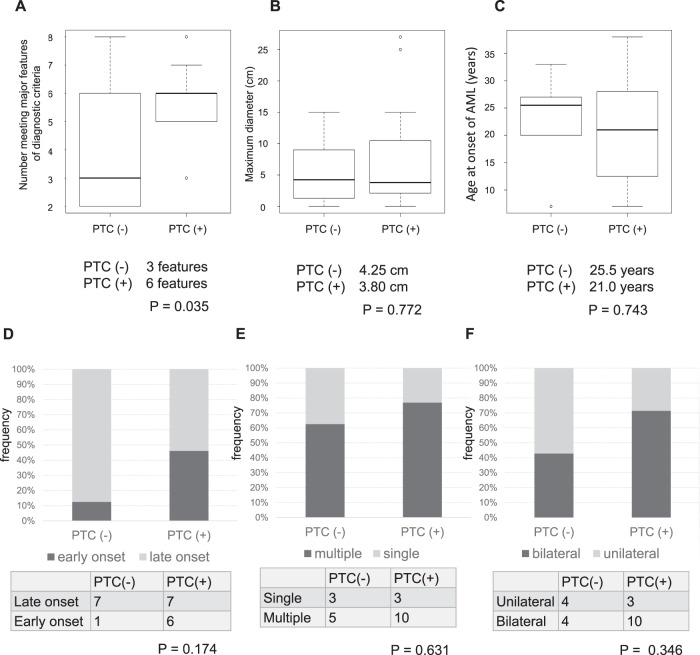
Phenotype of *TSC2*. **A** The number of positive major features listed in the diagnostic criteria^18^. Patients with a PTC were significantly more likely to manifest the major symptoms of the diagnostic criteria than those without a PTC. **B** There was no significant difference in the maximum diameter of AML with or without a PTC. **C** Although there was no statistically significant difference in the age of onset of AML between patients with and without a PTC, there was a tendency for patients with a PTC to develop AML at a younger age. **D** An onset under 20 years of age was defined as early onset, while an onset over 20 years of age was defined as late onset. There was no statistically significant difference, but the percentage of early onset was higher in patients with a PTC. **E**, **F** Although not statistically significant, the percentage of multiple and bilateral cases was higher in patients with a PTC.

No difference in AML diameter between patients with and without PTC mutations in *TSC2* was observed (Fig. 2B). Regarding the other clinical parameters, a lower age at onset was noted for patients with PTC mutations; however, the differences calculated using the Mann–Whitney *U*-test were not significant (Fig. 2C). Moreover, a higher incidence of early onset (onset before 20 years old) was observed in patients with PTC mutations than in patients with non-PTC mutations (Fig. 2D). Furthermore, the presence of multiple and bilateral tumors was more frequent in patients with PTC mutations, but the differences were not statistically significant (Fig. 2E, F).

## Discussion

In this study, we performed a genetic analysis of the *TSC1* or *TSC2* genes in our cohort of 30 patients with TSC. Genetic testing alone is not generally used for diagnostic purposes for patients with TSC, since diagnosis is typically based on clinical findings. If a pathological gene mutation is detected, it is a definitive diagnosis. However, if a pathological gene mutation is not detected, no definitive diagnosis can be made^17^. Therefore, genetic diagnosis is not always necessary or useful in renal AML caused by TSC. This is because the genotype-phenotype correlation in TSC is not yet fully understood. Previous studies on TSC genotypes and phenotypes have suggested that the symptoms of patients with *TSC2* mutations are more severe than those of patients with *TSC1* mutations^5,20–22^. However, there has been no correlation between mutation types reported.

In previous studies, disease severity was assessed in terms of overall incidence and tumor size^5,13^. Tumors in TSC have been suggested to be caused by the inactivation of both alleles of either *TSC1* or *TSC2* genes, consistent with Knudson’s two-hit tumor suppressor gene model. Although second-hit mutations are not always observed in each TSC lesion, most tumors in the brain or kidney have second-hit mutations^10,23^. In other words, a second hit is a necessary step for the development of tumors in TSC, and it may be assumed that somatic mutations play a pivotal role in the development of lesions. It is likely that factors other than the type of germline mutation in the *TSC1* or *TSC2* gene (i.e., modifier genes or environmental factors) may have influenced the incidence or size of tumors. Moreover, the data on the correlation between the type of mutations and the clinical course of the patient might be potentially confounded by whether the mutation was on the *TSC1* or *TSC2* gene since all the mutations in *TSC1* in this study were PTC mutations (Fig. 1).

In this study, we evaluated not only the frequency and diameter of AML but also the age of onset and occurrence of unilateral or bilateral tumors. We presumed that the evaluation of tumor size may have been incorrect in previous studies^5,13^ because tumor diameter is greatly affected by the timing of image evaluation. For this study, tumor diameters were measured based on the first image of the AML diagnosis to align the timing of image evaluation. However, no significant difference in AML diameter was observed between patients with or without PTC mutations. Although the age at onset was lower and the presence of multiple and bilateral tumors was more frequent in patients with PTC mutations, the differences were not statistically significant.

There are two major viewpoints regarding the weak but possible genotype-phenotype correlation. First, despite the effect of the second hit, the majority of second hits in TSC tumors are copy number neutral loss of heterozygosity (CN-LOH). The second viewpoint states that the weak correlation is because of the presence of few missense mutations in the second hit of the tumor^10,12^. Therefore, it is presumed that the residual activity of TSC protein in tumor cells is not affected by the second hit and is dependent on the type of germline mutant allele. If the germline mutation does not contain PTCs, the germline allele will produce tuberin that potentially has partial residual protein activity that may inhibit the mTOR pathway and suppress cell growth. In contrast, tuberin is not produced by NMD in the presence of a PTC. The loss of tuberin activity in cells with mutations with PTCs promotes faster cell growth, which may result in a relatively shorter time between tumor growth and detection. This could potentially result in a lower age of onset and the occurrence of bilateral and multiple tumors.

An alternative idea is that the first hit alone could possibly affect the cellular function responsible for the genotype-phenotype correlation. It is possible that the haploinsufficiency caused by germline mutations with a PTC reduces the amount of tuberin in the cells. This could prevent mTOR inhibition and accelerate the tumor growth of AMLs to a detectable size on imaging at an early stage. In addition, multiple and bilateral AML might be more common in patients with mutations containing a PTC on imaging at diagnosis, which is consistent with PTC-promoting tumor growth. Since multiple AMLs that develop in TSC are due to independent second-hit mutations in TSC1 or TSC2 that occur in different progenitor cells^12^, it is possible that a PTC affects the susceptibility of patients to somatic mutations. Other results showed that PTC-containing mutations were significantly more likely to be associated with SEN, cortical tubercles, and renal cysts. A cortical tuber is less likely to have a second hit, suggesting that monoallelic inactivation was sufficient and that the first hit had a strong influence on the phenotype. Our results might suggest that the presence of a PTC in germline mutations alone facilitated the development of some tumors in TSC.

This study has some limitations. The first was the small sample size, which may have been the reason why no statistically significant difference was observed. Since the sample size was small, the inclusion of exceptional cases, such as cases with a rare second hit, might affect the phenotype. This may be another reason for the lack of significant differences. Another limitation was the benign nature of the disease, making it difficult to collect samples from tumor lesions and assess the function of proteins in the lesions. The third limitation was case selection bias owing to the retrospective nature of the study.

In summary, TSC patients with PTC-producing mutations might potentially manifest more severe TSC phenotypes than those with missense mutations. The genetic diagnosis of TSC might be helpful in predicting the severity of TSC disease. A larger-scale study including the functional analysis of the variants deserves further investigation.

## Supplementary information

Supplemental Table
