# Supplementary material for: Genotype-phenotype correlation of renal lesions in the tuberous sclerosis complex
Source: Hum Genome Var. 2022 Feb 10;9:5. doi: 10.1038/s41439-022-00181-1 (PMC8831580; doi:10.1038/s41439-022-00181-1)
Supplement: Supplementary file 1 — Supplemental Table [file 41439_2022_181_MOESM1_ESM.docx]

Supplemental Table

Variants identified in this study

| Patient | Gene | Variant type | Nucleotide changes | Predicted amino acid changes |
| --- | --- | --- | --- | --- |
| TSC2-1 | *TSC2* | Missense | c.242T>G | p.Leu81Arg |
| TSC2-2 | *TSC2* | Missense | c.2150T>G | p.Leu717Arg |
| TSC2-3 | *TSC2* | Missense | c.4960G>A | p.Gly1654Ser |
| TSC2-4 | *TSC2* | Missense | c.5241C>A | p.Ala1712Asp |
| TSC2-5 | *TSC2* | Missense | c.5228G>A | p.Arg1743Gln |
| TSC2-6 | *TSC2* | Missense | c4493G>A | p.Ser1498Asn |
| TSC2-7 | *TSC2* | In-frame deletion | c.1093_1095delATC | p.Ile365del |
| TSC2-8 | *TSC2* | In-frame deletion | c.5238_5255del18 | p.His1746_Arg1751del |
| TSC2-9 | *TSC2* | Splicing | c1717-1G>A | N.D. |
| TSC2-10 | *TSC2* | Splicing | c.2742+1G>A | N.D. |
| TSC2-11 | *TSC2* | Frameshift | c724_725insA | p.Thr242AsnfsTer96 |
| TSC2-12 | *TSC2* | Frameshift | c.744_745insT | p.Val249CysfsTer89 |
| TSC2-13 | *TSC2* | Frameshift | c.826_827delAT | p.Met276ValfsTer7 |
| TSC2-14 | *TSC2* | Frameshift | c.826_827delAT | p.Met276ValfsTer61 |
| TSC2-15 | *TSC2* | Frameshift | c.34447delC | p.Leu1150TrpfsTer41 |
| TSC2-16 | *TSC2* | Frameshift | c.4219_4220insG | p.Val1407GlyfsTer7 |
| TSC2-17 | *TSC2* | Frameshift | c.4504delC | p.Leu1502CysfsTer74 |
| TSC2-18 | *TSC2* | Frameshift | c.4624delA | p.Thr1542ProfsTer34 |
| TSC2-19 | *TSC2* | Nonsense | c.645_646insTA | p.Glu216Ter |
| TSC2-20 | *TSC2* | Nonsense | c.912G>A | p.Trp304Ter |
| TSC2-21 | *TSC2* | Nonsense | c.1372C>T | p.Arg458Ter |
| TSC2-22 | *TSC2* | Nonsense | c.1513C>T | p.Arg505Ter |
| TSC2-23 | *TSC2* | Nonsense | c.5206_5207insA | p.Tyr1736Ter |
| TSC1-1 | *TSC1* | Frameshift | c.1888_1891delTTTC | p.Lys630GlnfsTer22 |
| TSC1-2 | *TSC1* | Nonsense | c1546C>T | p.Gln516Ter |
| TSC1-3 | *TSC1* | Nonsense | c.2283C>A | p.Tryr761Ter |
| TSC1-4 | *TSC1* | Nonsense | c.2716C>T | p.Gln906Ter |
| NMI-1 | None | None | None | None |
| NMI-2 | None | None | None | None |
| NMI-3 | None | None | None | None |

N.D. not determined
